# Life Cycle Assessment of River Sand and Aggregates Alternatives in Concrete

**DOI:** 10.3390/ma16052064

**Published:** 2023-03-02

**Authors:** Le Hung Anh, Florin-Constantin Mihai, Anna Belousova, Radek Kucera, Klaus-Dieter Oswald, Wolfgang Riedel, Naveedh Ahmed Sekar, Petra Schneider

**Affiliations:** 1 Institute for Environmental Science, Engineering & Management, Industrial University of Ho-Chi-Minh City, Ho Chi Minh City 700000, Vietnam; 2 Environmental Research Center “CERNESIM”, Department of Exact Sciences and Natural Sciences, Institute of Interdisciplinary Research, “Alexandru Ioan Cuza” University of Iasi, Bulevardul Carol I 11, 700506 Iași, Romania; 3 C&E Consulting und Engineering GmbH, Jagdschänkenstr. 52, 09117 Chemnitz, Germany; 4 Department for Water, Environment, Civil Engineering and Safety, University of Applied Sciences Magdeburg-Stendal, 39114 Magdeburg, Germany

**Keywords:** river sand alternatives, substitutive building materials, ecological footprint

## Abstract

Urbanization processes in Asia are still ongoing; thus, aggregate demand is expected to increase in following years. Even though construction and demolition waste is a source for secondary building materials in industrialized countries, it is not yet an alternative construction material source in Vietnam as the urbanization process is still ongoing. Thus, there is a need for river sand and aggregates alternatives in concrete, namely manufactured sand (m-sand) from primary solid rock materials and secondary waste materials. The focus in the present study for Vietnam was on m-sand sand as alternative for river sand, and different ashes as alternatives for cement in concrete. The investigations comprised concrete lab tests according to the formulations of concrete strength class C 25/30 in accordance with DIN EN 206, followed by a lifecycle assessment study in order to identify the environmental impact of the alternatives. In total 84 samples were investigated, consisting of 3 reference samples, 18 samples with primary substitutes, 18 samples with secondary substitutes, and 45 samples with cement substitutes. This kind of holistic investigation approach comprising material alternatives and accompanying LCA was the first study for Vietnam, and even for Asia, and represents a substantial added value for future policy development in order to cope with resource scarcity. The results show that with the exception of metamorphic rocks, all m-sands meet the requirements for quality concrete. In terms of cement replacement, the mixes showed that a higher percentage of ash reduces the compressive strength. The compressive strength values of the mixes with up to 10% coal filter ash or rice husk ash were equivalent to the C25/30 standard concrete formulation. Higher ash contents up to 30% lead to the reduction of the concrete quality. The LCA study’s results highlighted the better environmental footprints across environmental impact categories in the 10% substitution material in comparison to the use of primary materials. The LCA analysis results showed that cement as a component in concrete holds the highest footprint. The use of secondary waste as alternative for cement provides significant environmental advantage.

## 1. Introduction

Natural resources such as water and river sand are extracted from freshwater environments to feed industrial applications around the world while water pollution, erosion, and destruction of habitats are related impacts. Sand river is a key material source for building sector including concrete industry in addition to gravel, cement, and water. However, unsound exploitation of such natural resources from river systems poses serious risks for ecosystems and nearby human settlements [1,2] including Southeast Asia.

The magnitude of river sand mining operations are underestimated by official statistics due to the unregulated markets and supply chains particularly in emerging economies [3]. New assessment based on satellite images of sand mining operations in Mekong fluvial system of Cambodia revealed that rates of extraction have increased from 24 in 2016 to 59 Mt in 2020 at a rate of ~8 Mt/yr^−1^ [4]. Another study argues that 42 Mm^3^/yr of sand (average) is extracted from Vietnamese Mekong Delta [5]. Remote sensing applications is expected to emerge in following years to monitor the resource extraction from fluvial systems around the globe [6].

Linear economy based on extraction of natural raw materials disregards the resource depletion risks and potential use of secondary materials in construction industry [7]. Despite the fact natural aggregates (such as river sand) are seen a cheap commodity to be extracted by private investors, the implications on freshwater ecosystems and human settlement must be properly monitored while extraction activities must follow standardized regulations in Vietnam [8]. Furthermore, urban expansion will increase the sand, crushed rocks, and gravel materials to produce more concrete in Vietnam by 2030 [9]. Other natural-based sand materials are taken into consideration for concrete fabrication such as dune sand [10] or sea sand to decrease the current demands of river sand resources [11]. Even still the need of primary construction materials (particularly m-sand) in Vietnam is much larger than the availability of secondary materials, the circular economy vision provides sustainable alternatives to the reliance on natural-based sand aggregate used in current concrete industries around the globe. River sand material substitution is tested in laboratory based studies [12] or using lifecycle assessment (LCA) research to reveal the technical and environmental challenges to secondary materials options [13]. The recent review studies show that optimum level of sand replacement with byproducts is 20% to keep the qualitative features of concrete [14]. The same proportion is suggested in the case of dredge sediment, where a multilevel approach was implemented involving laboratory experiments and LCA to provide a sustainable use of sediment in concrete production [15]. 

Previous studies reveal waste foundry sand as alternative source to natural-based sand [16,17]. A review paper reveals that five industrial byproducts (coal bottom ash, copper slag, waste granite dust, waste glass cullet, palm oil clinker) have own features related to various concrete properties that must be further investigated to increase the substitution rate of river sand [18]. In addition to palm oil clinker, sugarcane bagasse ash is another organic material that could be used as a replacement of sand in foam concrete [19]. A major substitution potential for cement in Asia is attributed to rice husk ah (RHA). RHA is produced during the controlled combustion of rice husks. The prominent pore structure is a very important property of the material. Due to the strong water absorption caused by the pores, the addition of RHA reduces the flowability, but also increases the viscosity and thus the sedimentation stability of concrete [20].

Rapid urbanization process and development of construction sector release huge amounts of construction and demolition waste (CDW) that are disposed in municipal landfills or uncontrolled disposal sites polluting the natural environment under linear economy mechanisms [21]. On the other hand, there is growing interest in using recycled aggregates from CDW flow as building materials replacing the natural extraction of sand or gravels from riverine ecosystems [22]. This replacement may refer to concrete production, but also to cement or mortar production [23]. Recycling of iron tailing sand used as fine aggregate plus replacement rate of recycled concrete of 50% derived from construction waste show good mechanical properties of concrete [24]. Moreover, other waste streams such as plastic or e-waste are examined as material alternatives in concrete production in line with circular economy principles [25,26]. 

Sustainable construction mainly aims to reduce the negative environmental impacts generated by the construction industry [27]. The utilization of secondary waste materials in construction applications provides advantage for waste management and resource management [28,29,30]. The sustainability footprint for concrete production has long been a subject of debate, and several types of research have been focused on the use of recyclable materials in an attempt to reduce the consumption of naturally mined components [31,32,33,34,35]. However, the laboratory experiments must be supported by LCA analysis to demonstrate the viability of recycled aggregates use in concrete production in terms of technical and environmental concerns. The results comparison between natural and recycled aggregates in terms of concrete production quality and environmental footprint are necessary to adjust the current practices [36]. Therefore, this paper aims to investigate the substitution material options to river sand in concrete production in Vietnam using lab tests of concrete specimens manufactured from alternative materials and supporting LCA analysis as a response to current river sand mining and waste management challenges. The lab tests comprised the testing of uniaxial compression strengths of the test specimens in order to evaluate the destruction risk of concrete made from substitutes. The investigation of the test specimens destruction risk is a proven methodology in literature for the assessment of material durability [37,38,39,40]. Moreover, the goal of this study is to compare the environmental footprint of conventional concretes with containing different alternative aggregates relevant to Vietnam.

## 2. Materials and Methods

### 2.1. Regulatory Framework for the Investigations

The production of concrete, the design and construction of concrete parts, the determination of properties and application rules in construction are regulated in the standard TCVN 7570:2006 “Aggregates for concrete and mortar—Specifications” [41]. The main requirements for alternative primary sand sources are laid down in the norm TCVN 9205:2012 “Crushed Sand for Concrete and Mortar” [42]. The Vietnamese Ministry of Construction tightened production guidelines to replace natural sand in construction. For this purpose, the Resolution No. 46/NQ-CP was drawn up, which proposed solutions to overcome the shortage of construction sand in some localities [43]. A regulatory requirement is the increase in the use of ash and slag as sand substitute materials within the framework of Decision No. 452/QD-TTg on “Approval of projects to promote the treatment and use of ash, slag, gypsum from chemical thermal power plants, fertilizers as raw materials for the manufacture of building materials and in construction work” [44]. The Vietnamese technical regulations for the production of concrete, including all relevant examination methods and monitoring regulations, are in principle comparable to the German / European standardization. Moreover, the requirements for the environmental assessment of building materials also correspond to both European and German standards. In particular, the life cycle assessment according to ISO 14040 (Vietnamese standard TVCN ISO 14040:2009 [45]) plays a fundamental role as an assessment tool and shall be used as part of the implementation of the Government Decree 09/2021/ND-CP on the management of building materials [46], and the Decision No. 1266/QD-TTg “Strategy for the development of building materials in Vietnam for the period 2021–2030 with a vision up to 2050” [47].

### 2.2. Overview on the Investigation Approach

The approach to the investigation was stepwise, comprising:

- Literature and material availability research to find feasible substitutes;
- Two step concrete testing investigations to evaluate the possibilities of substituting sand with crushed sand or mineral wastes as well as cement with various ashes, comprising (a) manufacturing of concrete specimens and (b) uniaxial compression strengths (UCS) tests on them;
- Lifecycle assessment (LCA) of the investigated alternatives.

Having in view the general material availability in Vietnam, being representative for other Asian countries as well, the focus was put on primary materials that are available as natural resources in Vietnam in order to produce m-sand (primary materials) as well as fine particle aggregates that origin from secondary sources like agricultural or industrial wastes for replacing cement. Concrete cubes were manufactured with the alternative materials, followed by a subsequent uniaxial compression test in order to verify the unconfined compressive strengths. Following materials were investigated:

*Potential primary m-sand substitutes:* Crushed solid rocks, particularly marble, amphibolite, granodiorite, basalt.

**Figure 1:**
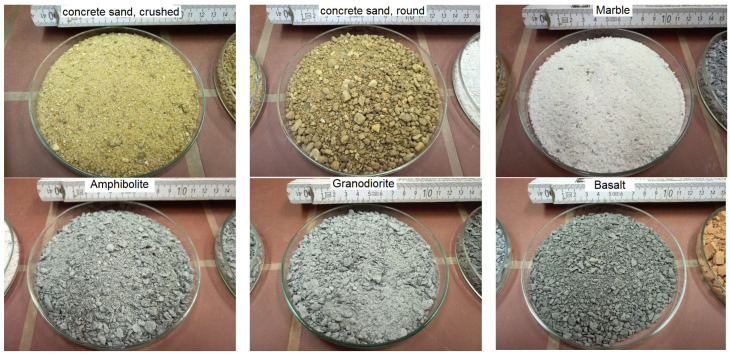
Potential primary sand sources crushed and round river concrete quartz sand and m-sand substitutes in form of rushed solid rocks: marble, amphibolite, granodiorite (a form of granite), and basalt (Figure source: Radek Kucera).

**Figure 2:**
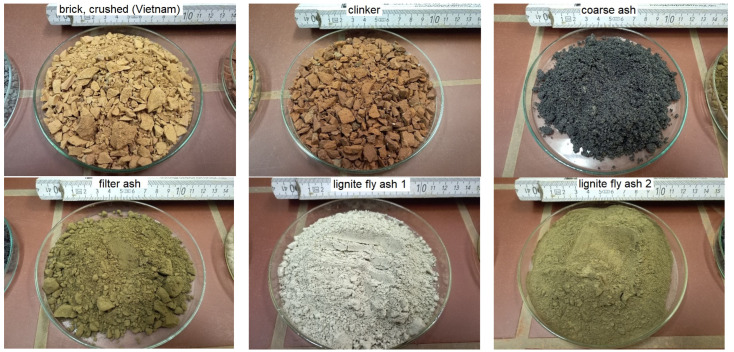
Potential secondary m-sand substitutes: crushed brick, crushed clinker; and secondary materials to substitute cement: lignite filter ash, coarse ash, and two types of fly ash (Figure source: Radek Kucera).

**Figure 3:**
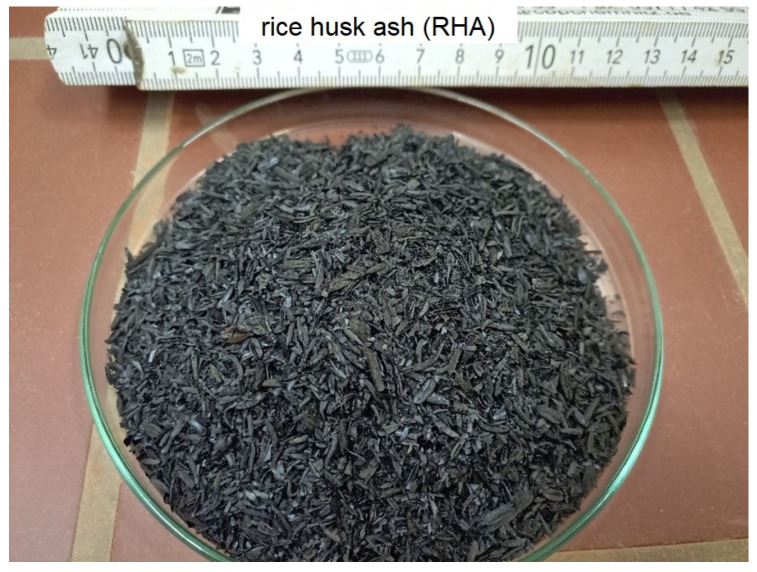
Potential secondary materials to substitute cement: rice husk ash (Figure source: Radek Kucera).

*Potential secondary m-sand substitutes:* Crushed brick, crushed clinker:

For m-sand comparison purposes was also used conventional crushed (broken) and river (round) concrete quartz sand (Figure 1), representing different processing steps. Moreover, following secondary materials to replace cement were tested (Figure 2 and Figure 3). The m-sand replacement materials were crushed with industrial crushers.

*Secondary materials to substitute cement:*

Lignite filter ash, coarse ash, fly ash, and rice husk ash.

Rice husk ash (RHA) was found in the literature research to be suitable for replacing (at least proportionally) cement in concrete [20,48,49,50,51,52,53]. A visual impression of RHA is shown in Figure 3.

### 2.3. Investigations for Substituting Sand with m-Sand or Mineral Wastes As Well As Cement with Various Ashes

The following basic standards were considered when conducting the laboratory tests:

- DIN EN 12390-1: Testing hardened concrete—Part 1: Shape, dimensions, and other requirements for specimens and molds [54]
- DIN EN 12390-2: Testing hardened concrete—Part 2: Making and curing specimens for strength tests [55]
- DIN EN 12620: Aggregates for concrete [56]
- DIN EN 206-1: Concrete—Part 1: Specification, performance, production, and conformity [57]
- DIN EN ISO 17892-7: Uniaxial compression strengths (UCS) test [58].

The concrete test specimens were produced according to the concrete formulations of strength class C 25/30 in accordance with DIN EN 206 [59]. For the calculation of the formulations, the grain density of the aggregates was determined according to DIN EN ISO 17892-3 [60]. A Portland cement CEM II/A-LL 32.5 R was used for testing the potential primary sand sources. Following nomenclature was used for the documentation: 

Primary aggregate substitutes: concrete sand, round (SR), concrete sand, broken (SB), marble (MA), amphibolite (AM), granodiorite (GD); basalt (BA);

Secondary aggregate substitutes: crushed brick (CB), crushed clinker (CC);

Secondary cement substitutes: lignite fly ash (LFA), coarse ash (CA), filter ash (FA) and rice husk ash (RHA).

Following test settings and series were performed, each test in triple repetition:

- Test series 1: concrete of strength class C 25/30 with the potential primary m-sand substitutes SR, SB, MA, AM, GD, BA, as well as a reference sample for comparison;
- Test series 2: concrete of strength class C 25/30 with CB 10%, 30%, 50%;
- Test series 3: concrete of strength class C 25/30 with CC in 10%, 30%, 50% share;
- Test series 4: concrete of strength class C 25/30 with LFA, CA, FA, and RHA in 10%, 20%, 30% share.

In total, 84 samples were investigated, consisting of 3 reference samples, 18 samples with primary substitutes, 18 samples with secondary substitutes, and 45 samples with cement substitutes. 

The base formulation C 25/30 concrete was replaced with different aggregates based on basalt, granite, marble, amphibolite, brick waste, and quartz (Table 1).

**Figure 4:**
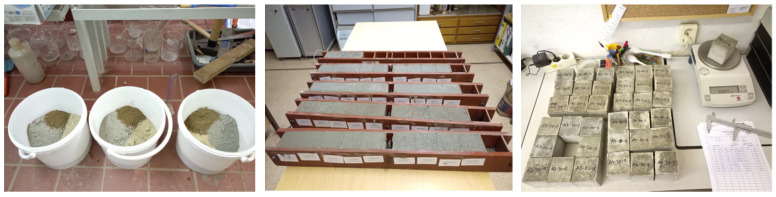
Manufacturing of the test specimens (Figure source: Radek Kucera).

Prior to testing, through particle size distribution analysis, it was investigated whether the concrete sand meets the requirements according to DIN EN 12620 [56] and DIN 1045-2 (requirements for concrete quality) [61]. The concrete test specimens were produced according to the strength class C 25/30 standard formulation according to DIN EN 206 [59]. For this purpose, 463 kg/m^3^ of cement and 1627 kg/m^3^ of aggregates (concrete sands or gravels) were mixed with 231 L/m^3^ of water. Figure 4 shows the manufacturing of the test cube specimens of 70 mm × 70 mm × 70 mm.

The second test series referred to the investigation of potential secondary m-sand substitutes, namely crushed brick and crushed clinker (Table 2 and Table 3).

The third test series referred to the investigation of secondary materials to substitute the cement though lignite filter ash, coarse ash, fly ash, and RHA (Table 4). The C 25/30 standard formulation should be reduced by the additions of 10%, 20%, and 30% ash. The reduction was carried out on four different ashes: coarse ash (ash 1), filter ash (ash 2), LFA 1 (ash 3), and LFA 2 (ash 4). The ashes are to replace the cement and, as such, the cement content. RHA was crushed in a ball mill due to its coarser particle size. The concrete specimens with ash contained crushed quartz as aggregate.

The specimens were stored at a temperature of approx. 20 °C. The specimens initially remained in the mold for 2 days. Subsequently, the already hardened specimens were removed from the mold and covered with a damp towel in a room at approx. 20 °C. The towels were wettened regularly to keep the relative humidity sufficiently constant. After 28 d curing time, the unconfined compressive strength of the specimens was determined according to DIN EN ISO 17892-7 [58] using the ZD 100 tensile/compressive testing machine (load up to 1000 kN). 

The specimens were firstly weighed, and the dimensions of the body were determined using a caliper gauge. The specimens were then subjected to stress under the ZD 100 tensile/compression testing machine, and the forces were noted until the respective specimen cube failed or cracked. The compressive strength was calculated from the parameters determined. The results are summarized in Section 3.

### 2.4. LCA of the Investigated Alternatives

The main objective was to estimate the environmental footprint of different sand and gravel alternatives utilized in selective construction applications replacing the conventional materials. In the present study was used lifecycle analysis (LCA) as a comparative sustainability performance tool. LCA is the most widely used holistic methodology, a multistage process, whose detailed definition is shown in the international standards of the series ISO 14040 [62] and ISO 14043 [63]. According to ISO 14040, LCA is defined as the “compilation and evaluation of the inputs, outputs and the potential environmental impacts of a product system throughout its life cycle”. Generally, LCA as a technique that is used to assess the environmental aspects associated with a product over its life cycle [64]. LCA has a structured four-stage framework:

- Goal and scope definition;
- Lifecycle inventory;
- Lifecycle impact assessment;
- Interpretation.

The LCA methodology is also recognized in Vietnam through the following norms:

- TVCN ISO 14040:2009: Environmental management—Product lifecycle assessment, principles, and framework;
- TVCN ISO 14041:2011: Life Cycle Assessment of Products—Principles and Framework;
- TVCN ISO/TR 14047:2018: Environmental Management—Life Cycle Assessment;
- TVCN ISO/TR 14048:2012: Environmental Management—Life Cycle Assessment—Data Documentation Format.

LCA was carried out using the Ecoinvent 3.6 database and the software Simapro 9.2. The software provided a user interface, the environmental information from the Ecoinvent database, and the options for the impact assessment method. The assessment methodology was ReCiPe 2016 Midpoint (H) V1.04/World (2010) H. The study involved the utilization of the LCA tool to calculate and analyze the environmental impacts of nonreinforced concrete of strength C25/30 using different aggregates with relevance to Vietnam. Using the LCA tool, the environmental footprints of the studied concrete were compared with each other. The system boundary considered was cradle to gate, where only material production and transportation to the site was considered. This study uses transport distances from cradle to gate as 30 km for traditional building materials based on information from Vietnamese experience. The functional unit for this study was the nonreinforced concrete sample of strength C25/30, where the materials were majorly produced outside of the construction area. The production of the materials comes with the product system. The functional unit for the study was a 1m^3^ block of concrete at strength C25 using different aggregates.

The selected unit processes for the LCI were modified for energy and water consumption, emissions for geographic relevance to the study. The cement unit process considered for this study was Portland cement global market-based because of Vietnamese relevant unit life cycle inventory process absence. The Ecoinvent Portland cement global unit process has a carbon footprint at a range of 0.44–0.96 kg CO_2_ eq. similar to the Vietnamese clinker and cement based on the environmental product declaration of the company INSEE Vietnam, Environmental Product Declaration (EPD) of Clinker and Cement VGD-ST-0014 [65]. 

In construction applications, the transportation of materials causes a substantial contribution to the environmental footprint [66,67,68]. Considering the information from Vietnamese experiences, a relatively short distance for material transportation is about 30 km for all materials in this study. The transport using Euro 4 type trucks with a payload capacity of 16–32 tons was assumed for all materials. Table 5 briefs the primary materials flow unit process in the concrete production for the chosen functional unit. The cement manufacturing, fine aggregate processing, and transportation inventory involve the generic global data from Ecoinvent. A transport distance of 30 km for building materials was considered. The unit processes used for concrete LCA from Ecoinvent are summarized in Table 5.

The LCA was completed using an energy demand scenario analysis that was carried out through the method “cumulative energy demand (CED) V1.11” in Ecoinvent, representing the direct and indirect energy use in the MJ unit throughout the life cycle. The method is based on higher heating values (HHV) [69]. The used impact categories are summarized in Table 6.

## 3. Results and Discussion

All lab results are documented in the Tables S1–S4 in the Supplementary Materials. The discussion of the data is provided below. The UCS results of the base formulation are provided in Table S1. The reached mean value σD concluded from 3 samples is 30.61 MPa.

### 3.1. Substituting Sand with m-Sand or Mineral Wastes As Well As Cement with Various Ashes

**Figure 5:**
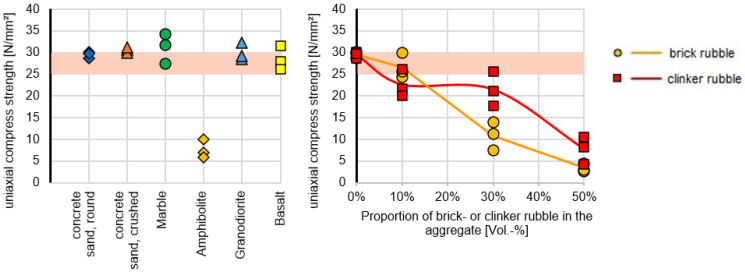
Results of unconfined compressive strengths of the base formulation with m-sand options.

The UCS results of the base formulation with primary m-sand substitutes are shown in Table S2 and Figure 5. The σD results for SR, SB, MA, BA, and GD ranged between 28.58 and 31.14 MPa. However, the σD results for AM reached the mean value of 7.56 MPa, proofing that amphibolite (AM) is not a feasible substitutive material for concrete fabrication. The UCS results of the base formulation with the secondary m-sand substitutes CC and CB are given in Table S3 and Figure 5. The mean σD results for CB were strongly dependent on the CB share in the specimens and covered a range between 3.42 (50% CB), 10,87 (30% CB), and 26.52 (10% CB) MPa and showed that CB is not a feasible substitute for manufacturing concrete. The mean σD results for CC were also strongly dependent on the CC share in the specimens and covered a range between 7.68 (50% CC), 21.50 (30% CC) and 22.65 (10% CC) Mpa and showed that CC is also not a feasible substitute for manufacturing concrete. 

Thus, the main result for aggregates replacement showed that except for amphibolite, brick, and clinker rubble, the C 25/30 standard formulations showed the required compressive strengths. The angular grains of the crushed materials do not have a substantial negative influence on the strength and the results are comparable to the concrete sand with round grain shape that represent the natural shape in contrast to the crushed materials. The substitutive material that resulted after the crushing process, showed visual differences in the grain form. Anyhow, all m-sand alternatives showed sand grains that have sharp edges and a rough surface. Moreover, all m-sand materials that originate from sedimentary and volcanic processes showed a compact material structure, in contrast to m-sand materials that originate from metamorphic formation processes. Those material have undergone a higher diagenetic pressure during the geological formation process leading to a platy to slatey structure and a higher tendency to break into plate-like particles. Consequently, the foliated grain shape of the metamorphic amphibolite leads to the significant reduction of the strength as was also reported by Małgorzata et al. (2016) [70]. The role of the grain structure of amphibolite as aggregate was also indicated by Anastasio et al. (2016) [71]. 

**Figure 6:**
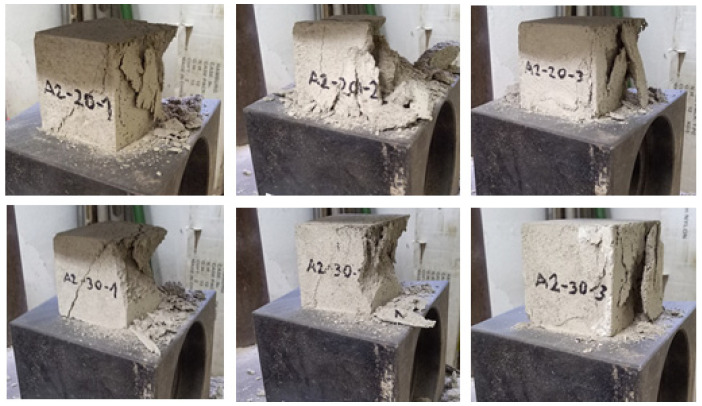
Impression of selected UCS specimens of the ash test series, here cubes having a filter ash share of 20 and 30%.

**Figure 7:**
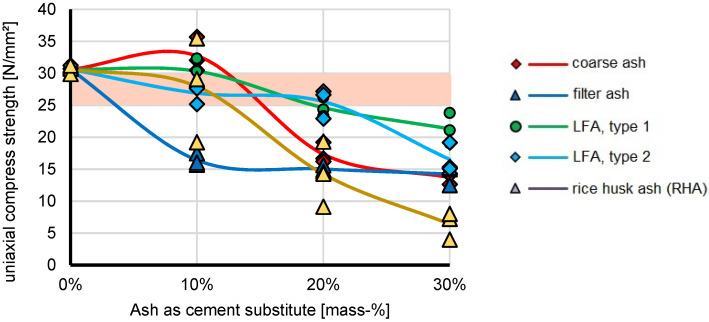
Results of unconfined compressive strengths of the ash test series.

The results for the base formulations for concrete of strength class C 25/30 with various ash shares as cement substitute in form of ashes are shown in Table S4 in the Supplementary Materials. An impression of selected UCS specimens of the ash test series are given in Figure 6, the graphic illustration of the results of unconfined compressive strengths of the ash test series is given in Figure 7.

The substitution of cement with ash was carried out with five different ashes: coarse Vietnam ash (CA), filter ash (FA), lignite filter ash in two grades (LFA1/2), and rice husk ash (RHA). It should be noted that different types of ash have very different hydraulic properties, if considered to be used as hydraulic binder instead of cement. The mean σD results for CA correlated with the CA share in the specimens and covered a range between 13.69 (30% CA), 17.35 (20% CA), and 32.72 (10% CA) MPa and showed that CA might be a feasible substitute for replacing cement, however, only up to a share of 10%. Moreover, the mean σD results for LFA correlated with the LFA1/LFA2 share in the specimens and covered a range between 21.31/16.52 (30% LFA), 24.63/25.57 (20% LFA) and 30.33/26.90 (10% LFA) MPa and underlined the variety of the LFA properties. Even a share of up to 10% might be added as cement substitute, there might be available certain LFA ashes that’s properties allow for a share of 20%. The variability of the properties was also indicated in Terzić et al. (2013) [72], who indicated lignite ash can be used as a component in cement, mortar, concrete, bricks, and tiles. It can be seen from the results that a higher ash content reduces the compressive strength. Formulations with 10% ash show higher compressive strengths than formulations with 20 or 30% ash. Compressive strength values were obtained for the formulations with 10% CA and LFA 1. The formulation with FA showed significantly lower σD values, namely 14.24 (30% FA), 15.03 (20% FA) and 16.41 (10% FA) MPa, than other formulations and is therefore not suitable for cement substitution/reduction. Generally, the mixes with 10% ash have higher strengths than mixes with 20% or 30% ash content. The results indicate that a higher ash content causes a lower the compressive strength. With the formulations containing 10% CA and LFA 1, the compressive strength values corresponding to the standard formulation were achieved. Four ashes still achieve 50% of the nominal compressive strength at 30% reduction. This is sufficient for simple buildings and substructures. RHA cannot be used for more than 10% cement substitution. Zaid et al. (2021) [73] concluded that more than 15% of cement with RHA will produce concrete with a low performance in terms of strength and durability. However, RHA might be a substitute in other forms of concrete such as lightweight concrete [48,49]. In any case, feasibility and property tests of the local ash are necessary before any application. The blends with FA and RHA showed significantly lower values than the other mixes and are therefore not suitable for cement reduction. According to Zaid et al. (2021) [73], the decreasing density correlates with an increase in the void content in concrete that causes less durable and lower strength as compared to normal concrete. The results of the compressive strength of the mixes showed that a higher percentage of ash reduces the compressive strength. The compressive strength values of the mixes with 10% CA and LFA 1 were equivalent to the strength of the C25/30 standard formulation. The results differ from the results of Sathawane et al. (2013) [50], who found a feasible share of 30%.

The addition of ash is only promising with 10% CA and LFA 1. Although the RHA and brick materials did not meet further expectations for use in concrete, these materials are well suited for landfill mineral seal layers in certain mix proportions [48].

Thus, with the exception of Amphibolite, all manufactured sands meet the requirements for quality concrete and achieve the required compressive strength as the formulations with natural concrete sand. The flaky grain shape of metamorphic amphibolite, however, leads to a significant reduction in strength. With manufactured aggregates from different rock types (mostly igneous or metamorphic), the same concrete quality can be achieved for all building construction purposes with good availability and short transport distances compared to river or sea dredging. Replacing 50% concrete sand with relatively soft brick fragments did not produce acceptable results. The brick grains absorb a lot of water. Anyhow, the German standard only permits a maximum of 30% brick content.

Magmatic and metamorphic rocks (with the exception of amphibolite) can be used as manufactured aggregates, replacing natural sand in concrete production. Selected ashes, especially coarse ash and lignite filter ash are suitable as cement substitutes, but only up to 10% proportion of concrete content. The ashes, but also brick dust, can also be used landfill mineral seal layers with up to 40% proportion of raw material content. Except for crushed amphibolite, brick rubble (crushed brick), and clinker rubble (crushed clinker), the modified C 25/30 concrete formulations show corresponding compressive strengths. The angular grains of the crushed materials obviously do not have a significant negative influence on the strength and the results are comparable to the concrete sand with round grain shape. The flaky grain shape of the metamorphic amphibolite leads to the significant reduction in strength. The brick and clinker rubble have negative effects on the strength in case they are substituted with higher percentages.

### 3.2. Lifecycle Assessment of the Investigated Alternatives

**Figure 8:**
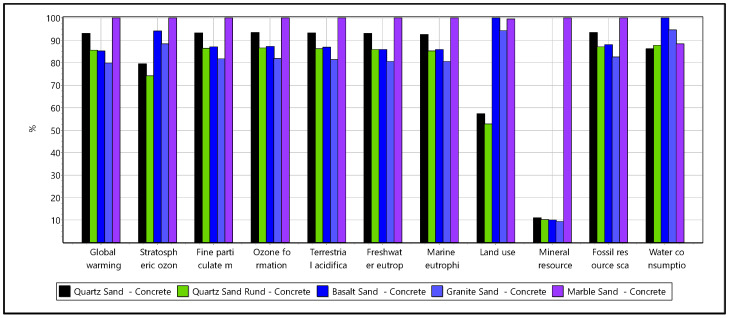
LCA comparison of primary raw material concrete samples across different impact categories.

For the LCA, amphibolite, clinker, and crushed brick were no longer considered, as those materials did not meet the concrete quality requirements. The LCA study results for the non-reinforced concrete of strength C25 involving conventional cement and alternative materials were compared and interpreted (Figure 8). Note that only the materials production or recycle process and their transportation to site impacts were reflected in the LCA calculations.

Among the different aggregate usage in the concrete, the granite-based concrete has the least impact with 346 kg CO_2_ eq per m^3^ of concrete. The granite-based concrete has a 20% lower GWP impact than marble based concrete. The basalt concrete and crushed rounded quartz concrete has a 15% lower GWP impact than marble concrete. The cement contributes the highest share of GWP about 95%. The remaining contribution arises from aggregate about 1.4–2.18% and transportation processes contributing 2.68–3.54%. The variation in the GWP contribution from aggregates was directly related to their mass present within the concrete mixes. Among the aggregates, the marble concrete utilizing limestone-based unit process shoed higher mineral resource scarcity. This could be based on the characterization values associated with limestone and the absence of such factors for other raw materials quartz, basalt, and granite. In general, the granite aggregate concrete had a lower mineral resource scarcity footprint of 3.73 kg Cu eq. The land use impact footprint remains low for quartz-based aggregates concrete, which was about 50–55% lower than other aggregate concrete. The water consumption impact remains marginally nearby for all the concrete samples ranging 3.12–3.64 m^3^, with granite aggregate concrete having a higher value. The marble concrete tends to have a higher footprint in acidification, eutrophication, and fossil resource scarcity impacts.

**Figure 9:**
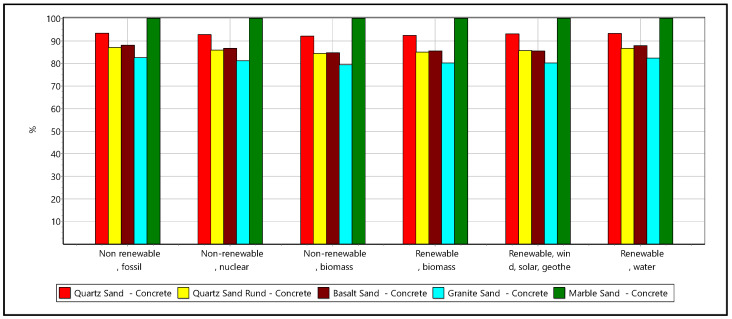
Energy demand analysis of primary raw material concrete samples within the life cycle of the concrete mixes.

The granite aggregate concrete has lower energy demand than other mixes across all the energy source categories (Figure 9). The energy demand reduction for basalt, quartz and granite aggregates based concretes varies between 7–18% than the marble aggregate concrete, same across all categories.

**Figure 10:**
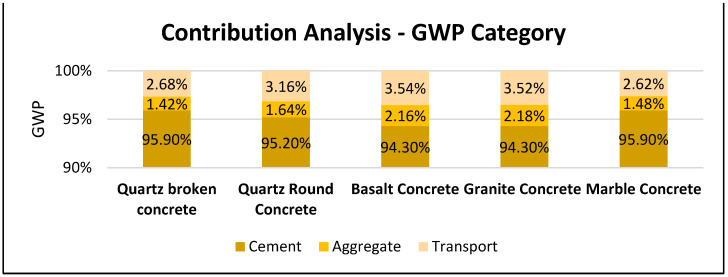
Global warming impact contribution analysis for primary raw material concrete samples.

Figure 10 illustrates the contributing units for the concrete sample mixes. The cement contributes the highest share of GWP about 95%.

The study’s results highlighted the better environmental footprints across impact categories in utilizing granite aggregate concrete. Despite granite aggregate lower values, all the aggregates in general only had marginal differences among the impact footprint. The LCA analysis results showed that cement as a component in concrete holds the highest impact footprint. Therefore, it becomes necessary to consider alternative materials with lower environmental footprint to replace cement or technological improvements along the cement value chain to lower the product footprint, as it is a widely used construction material. The LCA study also indicates the need for shifting the energy sources from fossil-based in the construction value chain to low-carbon or emission-based renewable sources to attain the global net-zero targets.

**Figure 11:**
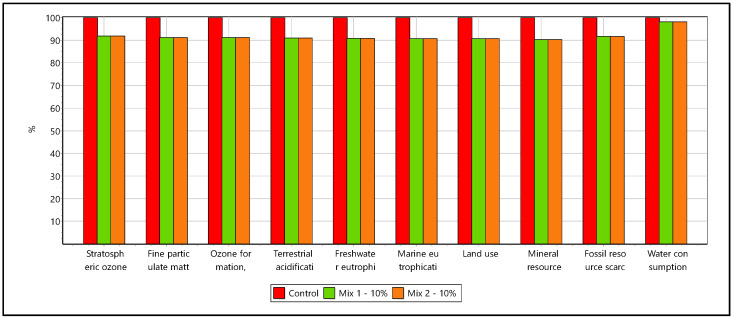
LCA comparison of secondary material cement replacement across different impact categories.

**Figure 12:**
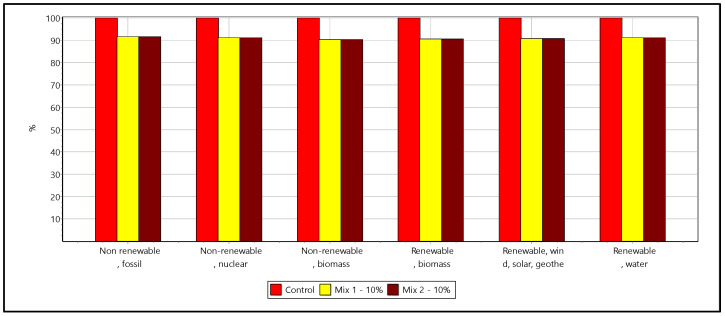
Energy demand analysis of secondary material cement replacement within the life cycle of the concrete mixes.

Figure 11 and Figure 12 show the LCA results for the cement replacement options. The cement replacement of 10% with ashes shows up a reduction of global warming potential (GWP) of about 38 kg per m3 of concrete for both the mixes. It has to be noted that the ashes carry a zero-allocation considered as waste, and the reduction is therefore directly contributed from 10% cement reduction. The GWP potential of the control sample recipe was 400 kg CO_2_ eq while the alternatives mixes had 362 kg CO_2_ eq (Figure 10).

The cement contributes the highest share of GWP about 95%. The remaining contribution arises from sand/aggregate about 1.5% and transportation processes contributing 3.17–2.71%. The alternative material utilization shows a reduction in mineral resource scarcity impact, highlighting a further possibility to research and improve the secondary waste material utilization in construction application at higher percentages.

Overall, the alternative mixes exhibit an average 10% reduction among all the impact categories except water consumption. In the water consumption category, the reduction of impact is only at marginal around 2%, which describes the different sources of energy demand involved within the life cycle of the concrete mixes. The alternative mixes have a 10% reduced energy consumption across the different energy sources (Figure 12). The investigation assessed the environmental impacts of utilizing ash-based alternative materials in the concrete application for replacing the cement consumption. The study’s results highlighted the better environmental footprints across impact categories in 10% substitution. LCA analysis is important to compare the use of natural vs recycled aggregates in terms of environmental impacts [74] in the context of adverse effects of river sand exploitation to natural ecosystems [75]. Further research and laboratory tests are required to develop scalable secondary material options for concrete industries [76,77] and to shift the paradigm towards a circular economy in the construction sector [78,79].

## 4. Conclusions

This study assessed the environmental impacts of utilizing different aggregate materials in concrete application. The key conclusion from the lab tests is that there are feasible primary and secondary raw materials in Vietnam that can be used for concrete production. The main findings can be summarized as follows:

Feasible primary m-sand substitutes for aggregates are crushed solid rocks, particularly with sedimentary and volcanic origin; metamorphic materials such as amphibolite are not feasible due to their reduced strength properties. Moreover, potential secondary m-sand substitutes like crushed brick and clinker do not meet the concrete quality requirements. Those materials might be used to produce m-sand to overcome resource scarcity.

Feasible secondary cement substitutes there were identified in this study are ashes from waste combustion processes. The results indicate that material mixes with up to 10% coal filter ash or rice husk ash were equivalent to the C25/30 standard concrete formulation. However, higher ash contents up to 30% lead to the reduction of the concrete quality.

The results underlined the availability of feasible materials for m-sand production in Vietnam. However, the use or primary materials causes a substantial environmental impact that was identified in the LCA investigation.

The LCA results highlighted the beneficial environmental footprints using substitutive building materials. However, as the urbanization and industrialization process is still ongoing in Vietnam, secondary material flows are not yet available to an extent to replace all primary raw materials.

This study’s results highlighted the better environmental footprints across impact categories in utilizing granite aggregate concrete. Despite granite aggregate lower values, all the aggregates in general only had marginal differences among the impact footprint. Therefore, on considering the uncertainty on the concrete mix analysis in this study, other aggregates can also be considered for sustainable consumption. The LCA analysis results showed that cement as a component in concrete holds the highest impact footprint. Therefore, it makes necessary to consider alternative materials with lower environmental footprint to replace cement or technological improvements along the cement value chain to lower the product footprint, as it is a widely used construction material. The LCA study also indicates the need for shifting the energy sources from fossil-based in the construction value chain to low carbon or emission-based renewable sources to attain the global net-zero targets.

Summarizing the results, the following needs as outlook were concluded.

*Improving information resources regarding construction materials demand using evidence-based methods:* To carry out planning for responsible mining of construction materials, an accurate estimate of the long-term supply and demand for these materials has to be made by the authorities. Without this important resource information, it is impossible to plan for sustainable consumption for a long-term period.

Promoting responsible consumption and secondary and renewable raw materials for construction along with their relevant quality requirements development: The shortage of river sand and other several construction materials based on the increasing demand will affect the construction industry greatly. Due to this reason, research must further be progressed for an easy and cheaply available alternative waste materials to replace the sand or several other fine aggregate materials completely or partially.

*Capacity of public authorities: Mineral products are essential for constructing modern societies and economies.* To meet the demands for construction materials extensive mining does not comply, rather ensuring natural environment protection and preservation is needed. The construction sector has extensive stakeholders. All stakeholders involved such as the provincial and local authorities, the mining industry, and the local population, must recognize that multiple interests exist. Subsequently, each entity must learn to pursue its own objectives in ways that move society, and thus the interests of all entities, forward as a whole.

*Focus to decarbonize the energy grid to reduce carbon intensity across value chain:* The study highlighted that the construction applications along their value chain involving construction materials extraction and transportation concerning Vietnam have a highly intensive fossil-based energy demand. Therefore, a focus on decarbonizing the energy grid in future holds a key in attaining net-zero emissions in coming years for Vietnam. A shift to increasing renewable-based energy could reduce the carbon footprint involved in construction material extraction, also within its value chain, and also bring benefits for electric mobility-based transportation.

*Considering switching to environmentally friendly transportation for construction materials:* The transportation of construction materials causes a substantial contribution of environmental and cost footprint within the construction activity lifecycle. Therefore, it is important to have an optimized transportation distance and transport mode for the construction materials.

*Further developing national and regional specific lifecycle inventory data for construction sector:* LCA is a beneficial tool in identifying the environmental burden of various products along within its value chain. The analysis helps to assess the negative impacts of inputs and outputs within the value chain on long-term sustainability and develop mitigation efforts to reduce the impacts across various points of the product lifecycle. The LCA benefits manufacturers to reduce the impact of their products, policymakers to plan strategically climate goal policies and consumers to be aware over the context of sustainable products. The “Vietnam Green Building Council (VGBC)—Green Database” (database for the environmental assessment of building materials to identify building materials with lower environmental impact) could be used as a starting point. 

## Figures and Tables

**Table 1:** Base formulations for concrete of strength class C 25/30 with potential primary m-sand substitutes.

		SR	SB	MA	AM	GD	BA
Cement	kg/m^3^	415	463	488	369	383	409
Water	L/m^3^	207	232	244	185	192	204
Additive	kg/m^3^	1.735	1.627	1.652	1.998	1.947	2.054
Grain density	g/cm^3^	2.65	2.65	2.78	2.89	2.86	3.12
k-Value		2.31	1.75	1.51	2.96	2.74	2.38

**Table 2:** Formulations for concrete of strength class C 25/30 with secondary m-sand substitutes: broken brick material.

		SB + CB 90:10 Vol.-%		SB + CB 70:30 Vol.-%		SB + CB 50:50 Vol.-%	
Cement	kg/m^3^	433		396		365	
Water	L/m^3^	216		198		182	
Additive	kg/m^3^	1477	169	1206	532	896	921
Grain density	g/cm^3^	2.65	2.73	2.65	2.73	2.65	2.73
k-Value		2.08				3.03	

**Table 3:** Formulations for concrete of strength class C 25/30 with secondary m-sand substitutes: broken brick material.

		SB + CC 90:10 Vol.-%		SB + CC 70:30 Vol.-%		SB + CC 50:50 Vol.-%	
Cement	kg/m^3^	435		401		371	
Water	L/m^3^	217		200		186	
Additive	kg/m^3^	1473	165	1198	517	888	894
Grain density	g/cm^3^	2.65	2.67	2.65	2.67	2.65	2.67
k-Value		2.06		2.49		2.92	

**Table 4:** Formulations for concrete of strength class C 25/30 with secondary materials to substitute cement through ash.

		Base	10% Ash	20% Ash	30% Ash
Cement	kg/m^3^	463	417	370	324
Ash	kg/m^3^	0	46	93	139
Water	L/m^3^	232	232	232	232
Aggregate	kg/m^3^	1627	1627	1627	1627

**Table 5:** Unit Processes used for concrete LCA from Ecoinvent.

Unit Process	Changes Made		Distance (km)
Cement, Portland {RoW}|production|APOS, U	Electricity, medium voltage {VN}|market for electricity, medium voltage|APOS, U	Electricity input changed to Vietnam region	30 km
(Crushed Quartz) Sand {RoW}|gravel and quarry operation|APOS, U	Sand, quartz Water, unspecified natural origin, VN Electricity, medium voltage {VN}|market for electricity, medium voltage|APOS, U	Input from nature changes to quartz Water and electricity input changed to Vietnam region	
(Basalt) Sand {RoW}|gravel and quarry operation|APOS, U	Basalt Water, unspecified natural origin, VN Electricity, medium voltage {VN}|market for electricity, medium voltage|APOS, U	Input from nature changes to basalt Water and electricity input changed to Vietnam region	
(Granite) Sand {RoW}|gravel and quarry operation|APOS, U	Granite Water, unspecified natural origin, VN Electricity, medium voltage {VN}|market for electricity, medium voltage|APOS, U	Input from nature changes to granite Water and electricity input changed to Vietnam region	
(Mable) Sand {RoW}|gravel and quarry operation|APOS, U	Limestone (proxy for marble) Water, unspecified natural origin, VN Electricity, medium voltage {VN}|market for electricity, medium voltage|APOS, U	Input from nature changes to limestone Water and electricity input changed to Vietnam region	
Tap water {RoW}|market for|APOS, U	Electricity, medium voltage {VN}|market for electricity, medium voltage|APOS, U Emissions—Water, VN	Water input changed to Vietnam region	
The market for transport, freight, lorry 16–32 metric ton, EURO4 RoW|APOS, U—30 km			

**Table 6:** Impact categories selected from ReCiPe and CED.

Category Group	Impact Category	Category Indicator
Climate change	Global warming potential	kg CO_2_ eq
Depletion of abiotic resources	Mineral resource scarcity	kg Cu eq
	Fossil resource scarcity	kg oil eq
Acidification	Terrestrial acidification	kg SO_2_ eq
Eutrophication	Freshwater eutrophication	kg P eq
Particulate matter	Fine particulate matter formation	kg PM2.5 eq
Ozone	Stratospheric ozone depletion	kg CFC11 eq
	Ozone formation terrestrial ecosystem	kg NOx eq
Cumulative energy demand	Cumulative energy consumption	KJ

## Data Availability

Not applicable.

## Supplementary Materials

The following supporting information can be downloaded at: https://www.mdpi.com/article/10.3390/ma16052064/s1, Table S1: Results of unconfined compressive strengths of the base formulation; Table S2: Results of the unconfined compressive strengths of the base formulation with potential primary m-sand substitutes; Table S3: Results of the unconfined compressive strengths of the base formulation with potential secondary m-sand substitutes; Table S4: Results of the unconfined compressive strengths of the base formulation with various ash shares as cement substitute.
