# Supplementary material for: Life Cycle Assessment of River Sand and Aggregates Alternatives in Concrete
Source: Materials (Basel). 2023 Mar 2;16(5):2064. doi: 10.3390/ma16052064 (PMC10004002; doi:10.3390/ma16052064)
Supplement: Supplementary file 1 [file materials-16-02064-s001.zip › materials-2216651-supplementary.pdf]

## Supplementary materials

**Table S1.** Results of unconfined compressive strengths of the base formulation.

| Basic Formulation | Specimen | Sample 1 | Sample 2 | Sample 3 |
|---|---|---|---|---|
| C 25/30 | Manufacture | 04.02.2021 | 04.02.2021 | 04.02.2021 |
| | UCS Testing | 05.03.2021 | 05.03.2021 | 05.03.2021 |
| | UCS [MPa] | 30.71 | 29.90 | 31.23 |
| | Mean Value $\sigma_D$ [MPa] | | 30.61 | |

**Table S2.** Results of the unconfined compressive strengths of the base formulation with potential primary m-sand substitutes.

| Formulation | Specimen | Base-SR-1 | Base-SR-2 | Base-SR-3 |
|---|---|---|---|---|
| C 25/30 | Manufacture | 10.12.2021 | 10.12.2021 | 10.12.2021 |
| SR | UCS Testing | 07.01.2022 | 07.01.2022 | 07.01.2022 |
| | UCS [MPa] | 30.11 | 28.72 | 29.80 |
| | Mean Value σD [MPa] | | 29.54 | |
| **Formulation** | **Specimen** | **Base-SB-1** | **Base-SB-2** | **Base-SB-3** |
| C 25/30 | Manufacture | 04.02.2021 | 04.02.2021 | 04.02.2021 |
| SB | UCS Testing | 05.03.2021 | 05.03.2021 | 05.03.2021 |
| | UCS [MPa] | 30.71 | 29.90 | 31.23 |
| | Mean Value σD [MPa] | | 30.61 | |
| **Formulation** | **Specimen** | **Base-MA-1** | **Base-MA-2** | **Base-MA-3** |
| C 25/30 | Manufacture | 06.12.2021 | 06.12.2021 | 06.12.2021 |
| MA | UCS Testing | 03.01.2022 | 03.01.2022 | 03.01.2022 |
| | UCS [MPa] | 27.46 | 34.27 | 31.69 |
| | Mean Value σD [MPa] | | 31.14 | |
| **Formulation** | **Specimen** | **Base-AM-1** | **Base-AM-2** | **Base-AM-3** |
| C 25/30 | Manufacture | 06.12.2021 | 06.12.2021 | 06.12.2021 |
| AM | UCS Testing | 03.01.2022 | 03.01.2022 | 03.01.2022 |
| | UCS [MPa] | 6.87 | 5.82 | 9.99 |
| | Mean Value σD [MPa] | | 7.56 | |
| **Formulation** | **Specimen** | **Base-GD-1** | **Base-GD-2** | **Base-GD-3** |
| C 25/30 | Manufacture | 06.12.2021 | 06.12.2021 | 06.12.2021 |
| GD | UCS Testing | 03.01.2022 | 03.01.2022 | 03.01.2022 |

| | | | | |
|---|---|---|---|---|
| | UCS [MPa] | 28.52 | 32.27 | 29.21 |
| | Mean Value σD [MPa] | | 30.00 | |
| **Formulation** | **Specimen** | **Base-BA-1** | **Base-BA-2** | **Base-BA-3** |
| C 25/30 | Manufacture | 10.12.2021 | 10.12.2021 | 10.12.2021 |
| BA | UCS Testing | 07.01.2022 | 07.01.2022 | 07.01.2022 |
| | UCS [MPa] | 31.62 | 28.04 | 26.08 |
| | Mean Value σD [MPa] | | 28.58 | |

**Table S3.** Results of the unconfined compressive strengths of the base formulation with potential secondary m-sand substitutes.

| **Formulation** | **Specimen** | **Sample 1** | **Sample 2** | **Sample 3** |
|---|---|---|---|---|
| C 25/30 | Manufacture | 13.04.2022 | 13.04.2022 | 13.04.2022 |
| CB + SB | UCS Testing | 11.05.2022 | 11.05.2022 | 11.05.2022 |
| 10 : 90 Vol.-% | UCS [MPa] | 24.45 | 25.53 | 29.94 |
| | Ø σD [MPa] | | 26.64 | |
| **Formulation** | **Specimen** | **Sample 1** | **Sample 2** | **Sample 3** |
| C 25/30 | Manufacture | 13.04.2022 | 13.04.2022 | 13.04.2022 |
| CB + SB | UCS Testing | 11.05.2022 | 11.05.2022 | 11.05.2022 |
| 30 : 70 Vol.-% | UCS [MPa] | 7.44 | 11.27 | 13.91 |
| | Ø σD [MPa] | | 10.87 | |
| **Formulation** | **Specimen** | **Sample 1** | **Sample 2** | **Sample 3** |
| C 25/30 | Manufacture | 13.12.2021 | 13.12.2021 | 13.12.2021 |
| CB + SB | UCS Testing | 10.01.2022 | 10.01.2022 | 10.01.2022 |
| 50 : 50 Vol.-% | UCS [MPa] | 2.71 | 3.05 | 4.49 |
| | Ø σD [MPa] | | 3.42 | |
| **Formulation** | **Specimen** | **Sample 1** | **Sample 2** | **Sample 3** |
| C 25/30 | Manufacture | 08.03.2022 | 08.03.2022 | 08.03.2022 |
| CC + SB | UCS Testing | 05.04.2022 | 05.04.2022 | 05.04.2022 |
| | UCS [MPa] | 21.65 | 20.07 | 26.22 |
| 10 : 90 Vol.-% | Ø σD [MPa] | | 22.65 | |
| **Formulation** | **Specimen** | **Sample 1** | **Sample 2** | **Sample 3** |
| C 25/30 | Manufacture | 08.03.2022 | 08.03.2022 | 08.03.2022 |
| CC + SB | UCS Testing | 05.04.2022 | 05.04.2022 | 05.04.2022 |
| 30 : 70 Vol.-% | UCS [MPa] | 21.13 | 17.72 | 25.65 |

| | | | | |
|---|---|---|---|---|
| | Ø σD [MPa] | | 21.50 | |

| Formulation | Specimen | Sample 1 | Sample 2 | Sample 3 |
|---|---|---|---|---|
| C 25/30 | Manufacture | 08.03.2022 | 08.03.2022 | 08.03.2022 |
| CC + SB | Testing | 05.04.2022 | 05.04.2022 | 05.04.2022 |
| 50 : 50 Vol.-% | UCS [MPa] | 8.27 | 4.17 | 10.59 |
| | Ø σD [MPa] | | 7.68 | |

**Table S4.** Results of the unconfined compressive strengths of the base formulation with various ash shares as cement substitute.

| | | | 10% Ash | | | 20% Ash | | | 30% Ash | |
|---|---|---|---|---|---|---|---|---|---|---|
| **Ash 1** | **Specimen** | **10%** | **10%** | **10%** | **20%** | **20%** | **20%** | **30%** | **30%** | **30%** |
| CA | Manufacture | 04.02.2021 | 04.02.2021 | 04.02.2021 | 04.02.2021 | 04.02.2021 | 04.02.2021 | 04.02.2021 | 04.02.2021 | 04.02.2021 |
| | UCS Testing | 05.03.2021 | 05.03.2021 | 05.03.2021 | 05.03.2021 | 05.03.2021 | 05.03.2021 | 05.03.2021 | 05.03.2021 | 05.03.2021 |
| | UCS [MPa] | 32,02 | 35,68 | 30,47 | 19,20 | 16,65 | 16,19 | 14,33 | 12,61 | 14,14 |
| | Ø σD [MPa] | | 32,72 | | | 17,35 | | | 13,69 | |
| **Ash 2** | **Specimen** | **10%** | **10%** | **10%** | **20%** | **20%** | **20%** | **30%** | **30%** | **30%** |
| FA | Manufacture | 04.02.2021 | 04.02.2021 | 04.02.2021 | 04.02.2021 | 04.02.2021 | 04.02.2021 | 04.02.2021 | 04.02.2021 | 04.02.2021 |
| | UCS Testing | 05.03.2021 | 05.03.2021 | 05.03.2021 | 05.03.2021 | 05.03.2021 | 05.03.2021 | 05.03.2021 | 05.03.2021 | 05.03.2021 |
| | UCS [MPa] | 15,70 | 17,49 | 16,05 | 14,88 | 15,60 | 14,61 | 12,50 | 14,98 | 15,25 |
| | Ø σD [MPa] | | 16,41 | | | 15,03 | | | 14,24 | |
| **Ash 3** | **Specimen** | **10%** | **10%** | **10%** | **20%** | **20%** | **20%** | **30%** | **30%** | **30%** |
| LFA 1 | Manufacture | 04.02.2021 | 04.02.2021 | 04.02.2021 | 04.02.2021 | 04.02.2021 | 04.02.2021 | 04.02.2021 | 04.02.2021 | 04.02.2021 |
| | UCS Testing | 05.03.2021 | 05.03.2021 | 05.03.2021 | 05.03.2021 | 05.03.2021 | 05.03.2021 | 05.03.2021 | 05.03.2021 | 05.03.2021 |
| | UCS [MPa] | 30,31 | 28,40 | 32,30 | 24,34 | 23,28 | 26,26 | 21,07 | 23,78 | 19,07 |
| | ØσD [MPa] | | 30,33 | | | 24,63 | | | 21,31 | |
| **Ash 4** | **Specimen** | **10%** | **10%** | **10%** | **20%** | **20%** | **20%** | **30%** | **30%** | **30%** |
| LFA 2 | Manufacture | 04.02.2021 | 04.02.2021 | 04.02.2021 | 04.02.2021 | 04.02.2021 | 04.02.2021 | 04.02.2021 | 04.02.2021 | 04.02.2021 |
| | UCS Testing | 05.03.2021 | 05.03.2021 | 05.03.2021 | 05.03.2021 | 05.03.2021 | 05.03.2021 | 05.03.2021 | 05.03.2021 | 05.03.2021 |
| | UCS [MPa] | 27,87 | 27,64 | 25,18 | 27,16 | 22,91 | 26,64 | 15,32 | 19,17 | 15,08 |
| | ØσD [MPa] | | 26,90 | | | 25,57 | | | 16,52 | |
| **Ash 5** | **Specimen** | **10%** | **10%** | **10%** | **20%** | **20%** | **20%** | **30%** | **30%** | **30%** |

| | | | | | | | | | | |
|---|---|---|---|---|---|---|---|---|---|---|
| RHA | Manufacture | 19.11.2021 | 19.11.2021 | 19.11.2021 | 19.11.2021 | 19.11.2021 | 19.11.2021 | 19.11.2021 | 19.11.2021 | 19.11.2021 |
| | UCS Testing | 17.12.2021 | 17.12.2021 | 17.12.2021 | 17.12.2021 | 17.12.2021 | 17.12.2021 | 17.12.2021 | 17.12.2021 | 17.12.2021 |
| | UCS [MPa] | 35,47 | 19,22 | 29,11 | 19,32 | 9,11 | 14,27 | 7,23 | 3,98 | 8,00 |
| | ∅σD [MPa] | | 27,94 | | | 14,23 | | | 6,40 | |
